# Evaluation of the Abdominal Aorta and External Iliac Arteries Using Three-Dimensional Time-of-Flight, Three Dimensional Electrocardiograph-Gated Fast Spin-Echo, and Contrast-Enhanced Magnetic Resonance Angiography in Clinically Healthy Cats

**DOI:** 10.3389/fvets.2022.819627

**Published:** 2022-06-09

**Authors:** Minju Lee, Minjung Ko, Jisoo Ahn, Jiyoung Ahn, Jin Yu, Jinhwa Chang, Sukhoon Oh, Dongwoo Chang

**Affiliations:** ^1^Section of Medical Imaging, Veterinary Medical Center, College of Veterinary Medicine, Chungbuk National University, Cheongju, South Korea; ^2^College of Veterinary Medicine, Western University of Health Sciences, Pomona, CA, United States; ^3^Korea Animal Medical Center, Cheongju, South Korea; ^4^Bio-Chemical Analysis Team, Korea Basic Science Institute, Daejeon, South Korea

**Keywords:** abdominal aorta, magnetic resonance angiography (MRA), arterial thromboembolism, feline, external iliac arteries

## Abstract

Arterial thromboembolism is associated with high morbidity and mortality rates in cats. Definitive diagnosis requires advanced imaging modalities, such as computed tomography angiography (CTA) and contrast-enhanced (CE) magnetic resonance angiography (MRA). However, CTA involves exposure to a large amount of ionized radiation, and CE-MRA can cause systemic nephrogenic fibrosis. Non-contrast-enhanced (NE) MRA can help accurately diagnose vascular lesions without such limitations. In this study, we evaluated the ability of NE-MRA using three-dimensional electrocardiograph-gated fast spin-echo (3D ECG-FSE) and 3D time-of-flight (3D TOF) imaging to visualize the aorta and external iliac arteries in clinically healthy cats and compared the results with those obtained using CE-MRA. All 11 cats underwent 3D ECG-FSE, 3D TOF, and CE-MRA sequences. Relative signal intensity (rSI) for quantitative image analysis and image quality scores (IQS) for qualitative image analysis were assessed; the rSI values based on the 3D TOF evaluations were significantly lower than those obtained using 3D ECG-FSE (aorta 3D TOF: 0.57 ± 0.06, aorta 3D ECG-FSE: 0.83 ± 0.06, *P* < 0.001; external iliac arteries 3D TOF: 0.45 ± 0.06, external iliac arteries 3D ECG-FSE:0.80 ± 0.05, *P* < 0.001) and similar to those obtained using CE-MRA (aorta: 0.58 ± 0.05, external iliac arteries: 0.57 ± 0.03). Moreover, IQS obtained using 3D TOF were significantly higher than those obtained using 3D ECG-FSE (aorta 3D TOF: 3.95 ± 0.15, aorta 3D ECG-FSE: 2.32 ± 0.60, *P* < 0.001; external iliac arteries 3D ECG-FSE: 3.98 ± 0.08, external iliac arteries 3D ECG-FSE: 2.23 ± 0.56, *P* < 0.001) and similar to those obtained using CE-MRA (aorta: 3.61 ± 0.41, external iliac arteries: 3.57 ± 0.41). Thus, 3D TOF is more suitable and produces consistent image quality for visualizing the aorta and external iliac arteries in clinically healthy cats and this will be of great help in the diagnosis of FATE.

## Introduction

Feline arterial thromboembolism (FATE) is a condition associated with high morbidity and mortality rates that is most commonly caused by underlying cardiomyopathy (1–4). The most frequent site of FATE is the terminal aorta (saddle thrombus) (5). Clinical signs are insufficient to produce a definitive diagnosis of FATE. A history of predisposing conditions might support diagnosis, but FATE should be differentiated from other diseases such as spinal cord diseases, including intervertebral disk disease, neoplasia, and embolism, and acute intracranial disorders, including embolism, trauma, and toxicity, that also cause acute loss of lower-extremity function.

Abdominal ultrasonography is useful for identifying large aortic thrombi. However, it requires technical expertise and can easily miss relatively small thrombi, especially when performed by an inexperienced sonographer. Thus, as immature thrombi appear hypoechoic and homogenous in acute conditions, FATE cannot be excluded even if a thrombus is not clearly observed (6, 7). Doppler flow assessments can usually help diagnose appendicular FATE. However, it is not possible to evaluate the full extent of an aortic thrombus because of difficulties in remaining perpendicular to the aorta and external iliac arteries. Moreover, if the vessels are partially occluded, the presence of arterial blood flow cannot be excluded (2). A definitive diagnosis thus requires advanced imaging modalities such as computed tomography and magnetic resonance imaging (MRI), which allow for the visualization and evaluation of the full extent of thrombi in the aorta and lower extremity arteries at high speed (8–10).

However, computed tomographic angiography (CTA) has the disadvantages of exposing the patient to a large amount of ionized radiation and causing contrast-induced nephropathy. Gadolinium-contrast enhanced (CE) magnetic resonance angiography (MRA) yields high sensitivity (81–99.5%) and high specificity (89–99%) for detecting significant artery stenosis in human patients (11, 12). Nevertheless, recent studies have demonstrated a correlation between gadolinium-based contrast media and nephrogenic systemic fibrosis in human patients with impaired renal function, suggesting that gadolinium causes damage by affecting the glomerular filtration rate (13, 14).

Recently, alternative non-contrast-enhanced (NE) MRA techniques have been widely used to evaluate the entire lower extremity arteries, with high accuracy for detecting vascular stenosis and thrombi without side effects in humans. It is possible to evaluate only the arteries by separating or suppressing venous signals from the artery signals. Furthermore, repetitive scans can be performed as many times as necessary because no contrast media is used (15, 16). Nevertheless, no previous veterinary studies have evaluated the aorta and external iliac arteries using NE-MRA to confirm thrombi in patients with FATE.

Electrocardiograph-gated three-dimensional fast spin-echo (3D ECG-FSE) sequence has been most frequently used during routine clinical examinations to evaluate thrombi and stenosis in the lower extremity arteries of humans. The T2-weighted 3D FSE technique using partial Fourier acquisition is based on cardiac gating with either ECG or peripheral pulse gated to the image arterial bright blood, with MR using the difference in arterial flow velocity during the systole and diastole (16–18). 3D ECG-FSE has been further improved to obtain a better display of slow-flowing blood, allow better visualization of collateral arteries, and reduce motion artifacts (10, 19, 20).

The 3D time-of-flight (3D TOF) method is still the dominant non-contrast method for neurovascular MRA. Stationary tissues become magnetically saturated by multiple repetitive radiofrequency (RF) pulses, whereas flowing blood does not experience these pulses. Therefore, initial magnetization is high. The signal from inflowing blood seems paradoxically bright compared to background tissues (16, 21).

We evaluated the ability of NE-MRA to visualize the feline aorta and external iliac arteries in clinically healthy cats and compared the results with those obtained using 3D ECG-FSE, 3D TOF NE-MRA, and gadolinium CE-MRA using 1.5-T MRI. We hypothesized that NE-MRA techniques could be used to visualize the aorta and external iliac arteries of clinically healthy cats. Furthermore, we hypothesized that 3D TOF, independent of the heart rate, would provide better a visualization of the aorta and external iliac arteries than 3D ECG-FSE, which can be influenced by tachycardia.

## Materials and Methods

### Study Design

This prospective analytical study was approved by the Chungbuk National University Institutional Animal Care and Use Committee. All protocols followed the Chungbuk National University Guidelines for Animal Experiments. Informed consent was obtained from all cat owners before study enrollment.

From June to July 2020, 11 cats owned by client were included in this study: one intact male, five castrated males, and five spayed females. The mean age of the cats was 41.9 (11–81) months, and the mean body weight was 4.50 (3.1–5.85) kg. The breeds were Domestic Shorthair (*n* = 10) and Norwegian Forest (*n* = 1) (Table 1). All cats were confirmed to be clinically healthy after thorough physical examination, blood analysis including complete blood cell count, serum biochemical analysis (total protein, albumin, alanine aminotransferase, alkaline phosphatase, blood urea nitrogen, creatinine, glucose, and C-reactive protein), analysis of electrolytes (Na, K, and Cl), urinalysis, and diagnostic imaging including thoracic radiography, abdominal radiography, abdominal ultrasonography, and echocardiography.

**Table 1:** Individual characteristics of eleven clinically healthy cats.

**Subject**	**Age (months)**	**Sex**	**Breed**	**Body weight (kg)**	**Used segments**
Cat 1	72	SF	DSH	4.2	Aorta, External iliac arteries
Cat 2	16	CM	DSH	5.85	Aorta, External iliac arteries
Cat 3	61	CM	DSH	4.25	Aorta, External iliac arteries
Cat 4	36	SF	DSH	4.2	Aorta, External iliac arteries
Cat 5	11	CM	NF	4.2	Aorta, External iliac arteries
Cat 6	66	SF	DSH	5.1	Aorta, External iliac arteries
Cat 7	11	IM	DSH	3.1	Aorta, External iliac arteries
Cat 8	54	SF	DSH	5.2	Aorta, External iliac arteries
Cat 9	81	CM	DSH	4.2	Aorta, External iliac arteries
Cat 10	22	CM	DSH	5	Aorta, External iliac arteries
Cat 11	31	SF	DSH	4.15	Aorta, External iliac arteries

*SF, spayed female; CM, castrated male; IM, intact male; DSH, Domestic Short hair; NF, Norway Forest*.

All cats were sedated with two separate intravenous injections of butorphanol (0.2 mg/kg; Butophan; Myung Moon Pharm, Hwaseong, Korea) and midazolam (0.1 mg/kg; Bukwang Midazolam; Bukwang Pharm, Ansan, Korea) and propofol (4 mg/kg intravenous injection; Provive injection 1%; Myung Moon Pharm) was subsequently injected for general anesthesia. Inhalation anesthesia was maintained with 2.5 to 3.0% isoflurane (Terrell; Piramal Critical Care, Inc., Bethlehem, PA, USA) and oxygen (1.0–1.2 L/min). The cats were kept intubated with a cuffed endotracheal tube and maintained under positive pressure ventilation using 100% oxygen with a peak inspired pressure of 8–12 mmHg at a respiratory rate of 7–15 breaths/min, tidal volume of 15–20 mL/kg, and end-tidal CO_2_ concentration of 35–45 mmHg. During general anesthesia, the heart rate, non-invasive systolic blood pressure, respiratory rate, end-tidal CO_2_ concentration, and isoflurane concentration were monitored continuously using a patient monitoring machine (IntelliVue MP70, PHILIPS, Bothell, Washington). Decisions regarding subject inclusion and exclusion for the study were made by two authors with diagnostic imaging expertise (M.J.L. and M.J.K.).

### MRA Techniques

**Figure 1:**
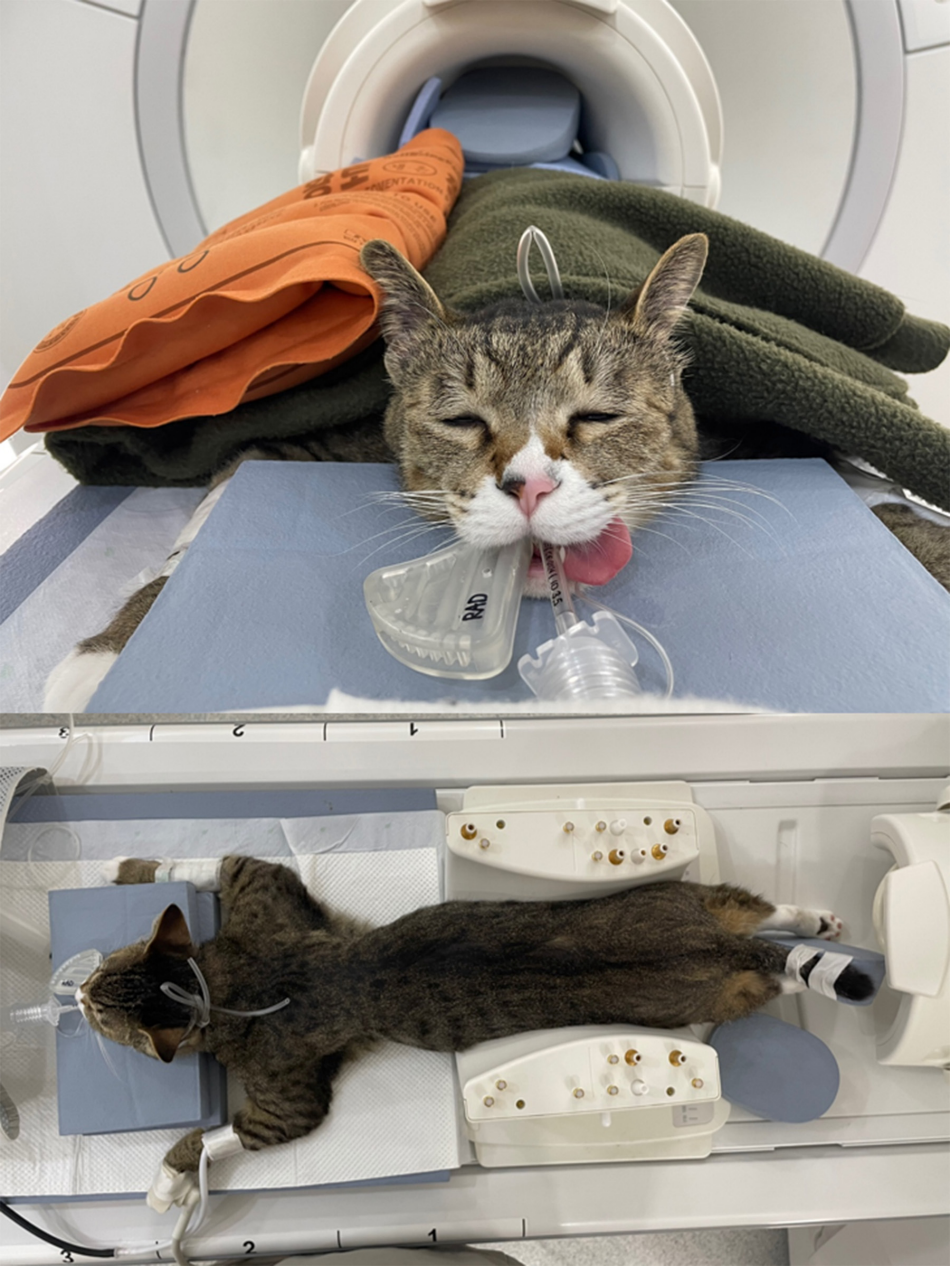
An eight-channel human knee coil with a 1.5-T magnet was used for signal reception. Under inhalational anesthesia, the cat was placed in the sternal recumbency position with the hind limbs caudally extended; the thigh region was included in the coil. To prevent the hind limbs from being disturbed, the sponge and hind limbs were fixed together and blankets and hot packs were applied over the cat to maintain its body temperature. MRI, magnetic resonance imaging.

MRI scans of all cats were performed using a 1.5-T magnet (SIGNA Creator; GE Healthcare, Milwaukee, WI, USA). All cats underwent general anesthesia with butorphanol, midazolam and propofol were ventilated, and were placed in the sternal recumbency position with the feet first in the scanner and hind limbs extended (Figure 1). An eight-channel knee coil was used for signal reception. Subsequently, 3D ECG-FSE and 3D TOF were performed in a random order immediately followed by gadolinium CE-MRA. The field of view was adjusted to include the abdominopelvic region from the left kidney to caudal thigh. We performed 3D ECG-FSE, 3D TOF and CE-MRA analyses in accordance with our parameters (Table 2). For 3D ECG-FSE, peripheral pulse gating was used to synchronize image acquisition and cardiac cycle. To determine optimal trigger delays, a dorsal single-shot fast spin-echo (SSFSE) sequence was performed in advance.

**Table 2:** Imaging parameters of 3D ECG-FSE and 3D TOF in non-contrast-enhanced magnetic resonance angiography (NE MRA) and contrast-enhanced magnetic resonance angiography (CE MRA).

**Imaging parameters**	**3D ECG-FSE**	**3D TOF**	**CE MRA (3D TOF FSPGR)**
Acquisition mode	3D	3D	3D
Acquisition time (minutes:seconds)	8–12 RR interval	8:24	1:24
TR (msec)	6,750	27	5.7
TE (msec)	60.4	6.8	1.6
Flip angle (degrees)	90	25	30
Field of View (mm)	240–260	240–260	240–260
Voxel size (mm)	1.0 ×0.9 ×1.2	0.8 ×1.1 ×1.2	1.0 ×0.9 ×1.2
Acceleration factor	N/A	Two	Two
Bandwidth (Hz/px)	83.33	25	50
Fat suppression	No	No	Yes
Thickness	1.2	1.2	1.2
Matrix	288 ×192	320 ×220	240 ×288

*The 3D ECG-FSE acquisition time was dependent on the subject's heart rate. RR interval: the time elapsed between two successive R waves of the QRS signal on the electrocardiogram*.

*N/A, not available; 3D ECG-FSE, three-dimensional echocardiograph-gated fast spin echo; 3D TOF, three-dimensional time-of-flight; FSPGR, contrast-enhanced 3D TOF fast spoiled gradient echo; TE, echo time; TR, repetition time*.

### Contrast-Enhanced MRA

In 11 cats, Gadolinium CE-MRA was performed using the 3D TOF fast spoiled gradient echo technique with the multiphase method. Each phase lasted 27 s, and four consecutive phases were performed. Before injecting the contrast medium, a precontrast scout scan was performed to check the anatomical coverage and to obtain the subtraction image from the postcontrast study. The scan was started at the same time as the intravenous bolus injection of 0.2 mmol/kg of Gadolinium DTPA (Clariscan® GE Healthcare AS, Oslo, Norway) with an auto-injector at a rate of 2 mL/s, followed by a 10 mL saline flush at the same rate. After subtraction processing using baseline images, 3D reconstructed MRA images were acquired through maximum intensity projection.

### MRA Image Analysis

All digital images were assessed using the Digital Imaging and Communications in Medicine (DICOM) viewer (RadiAnt DICOM Viewer software; Medixant Co., Poznan, Poland). Measurements were performed using electronic calipers and the same imaging viewer. Post-processing and maximum-intensity projection images of the subtracted data sets were analyzed using a workstation (Volume viewer 14.0 Ext.2; GE Healthcare, Milwaukee, WI, USA). Images obtained during all examinations were evaluated twice, separately, and in random order by two observers with diagnostic imaging expertise (M. J. L. and M. J. K.) who were blinded to patient signaling and sequence information. Disagreements were resolved through consensus.

### Quantitative Image Analysis

For the quantitative image analysis of clinically healthy cats, the signal-to-noise ratio (SNR) could not be calculated because the parallel imaging used in 3D TOF caused heterogeneous noise distribution (22).

Signal intensity (SI) was calculated at the center of the normal portion of each arterial segment with circular regions of interest (ROI) of 0.01–0.02 mm^2^. The rSI of the surrounding stationary soft tissue was measured from the muscle next to the ROI in the vessel with an area of 0.05–0.09 mm^2^. The relative signal intensity (rSI) was determined for the quantitative assessment of the relative contrast for each arterial segment, including the aorta and external iliac arteries of the bilateral extremities (22).


$$rSI=\frac{Artery\;SI-Adjacent\;Muscle\;SI}{Artery\;SI+Adjacent\;Muscle\;SI}$$

### Qualitative Image Analysis

**Figure 2:**
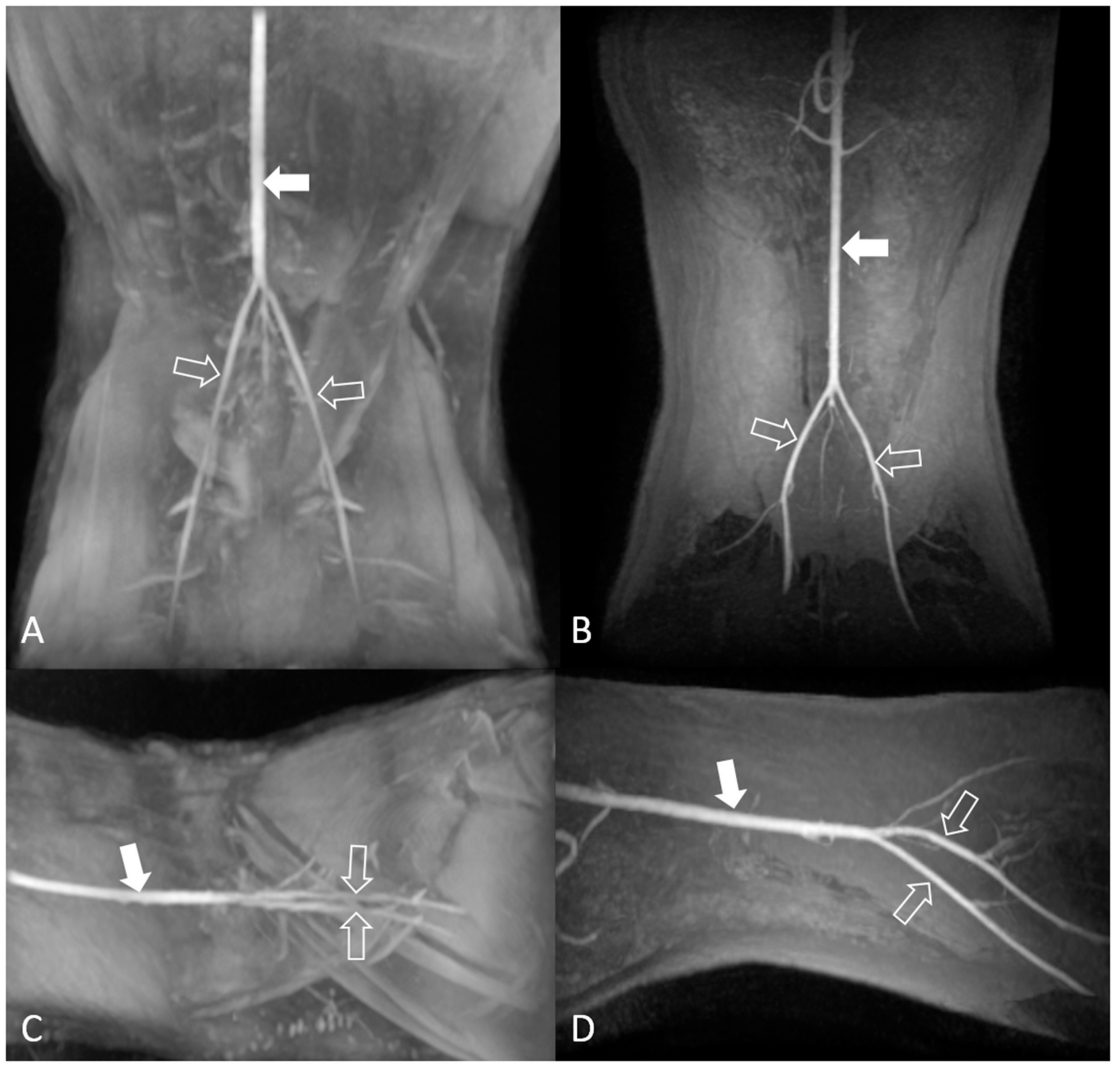
Maximum-intensity projection reconstruction of non-contrast-enhanced 3D TOF magnetic resonance angiography images of the dorsal view **(A)** and lateral view **(C)**, and contrast-enhanced 3D TOF fast spoiled gradient-echo magnetic resonance angiography images of the dorsal view **(B)** and lateral view **(D)** of the feline aorta and external iliac arteries at 1.5 Tesla. The solid arrow indicates the aorta, and the open arrows indicate the external iliac arteries. 3D TOF, three-dimensional time-of-flight.

The image quality for the dorsal maximum-intensity projection reconstruction of the aorta and external iliac arteries was assessed by turning the image by 360° (Figure 2). Two observers independently ranked quality using a four-point assessment scale based on the delineation of artery borders, venous contamination, and artifacts (Table 3). A grade score of 2–4 was deemed diagnostic (17).

**Table 3:** Image quality assessment in the qualitative image analysis.

**Score**	**Grade**	**Description**
1	Poor	Non-diagnostic due to poor delineation of major arteries or severe venous contamination
2	Fair	Fair delineation of major arteries or some venous contamination
3	Good	Good delineation of major arteries or minor venous contamination
4	Excellent	Excellent delineation of major arteries and no venous contamination

### Statistical Analysis

The analyses were performed using the statistical software SPSS for Windows (version 21.0; SPSS Inc., Chicago, IL, USA) and Prism 9.0 (GraphPad Software, San Diego, CA, USA). Data for each segment of the aorta and external iliac arteries are expressed as mean ± standard deviation and continuous numerical variables. For all tests, statistical significance was set at *P* < 0.05. The rSI values of the 3D ECG-FSE group and 3D TOF group were obtained, and data were analyzed by one-way analysis of variance (ANOVA) procedure after evaluating normality via the Shapiro–Wilk test. The image quality scores (IQS) of 3D ECG-FSE and 3D TOF were compared using non-parametric tests, Spearman's rank correlation and simple regression analysis. Spearman's rank correlation coefficients and simple regression analysis were calculated for IQS and various variables, including heart rate, systolic blood pressure, respiratory rate, and scan time, to determine whether any individual factor associated with general anesthesia correlated more closely with the IQS of the 3D ECG-FSE and 3D TOF at the time of scanning for each cat. For all analyses, a significant difference was defined as *P* < 0.05. To evaluate the reproducibility and consistency of rSI and IQS between the two observers, intraclass correlation coefficients were calculated using a two-way mixed model. Generally, it can be evaluated as a good degree of consistency if it is 0.75 or higher. Lastly, Fisher's exact test was used to confirm the association between sequence and IQS.

## Results

**Figure 3:**
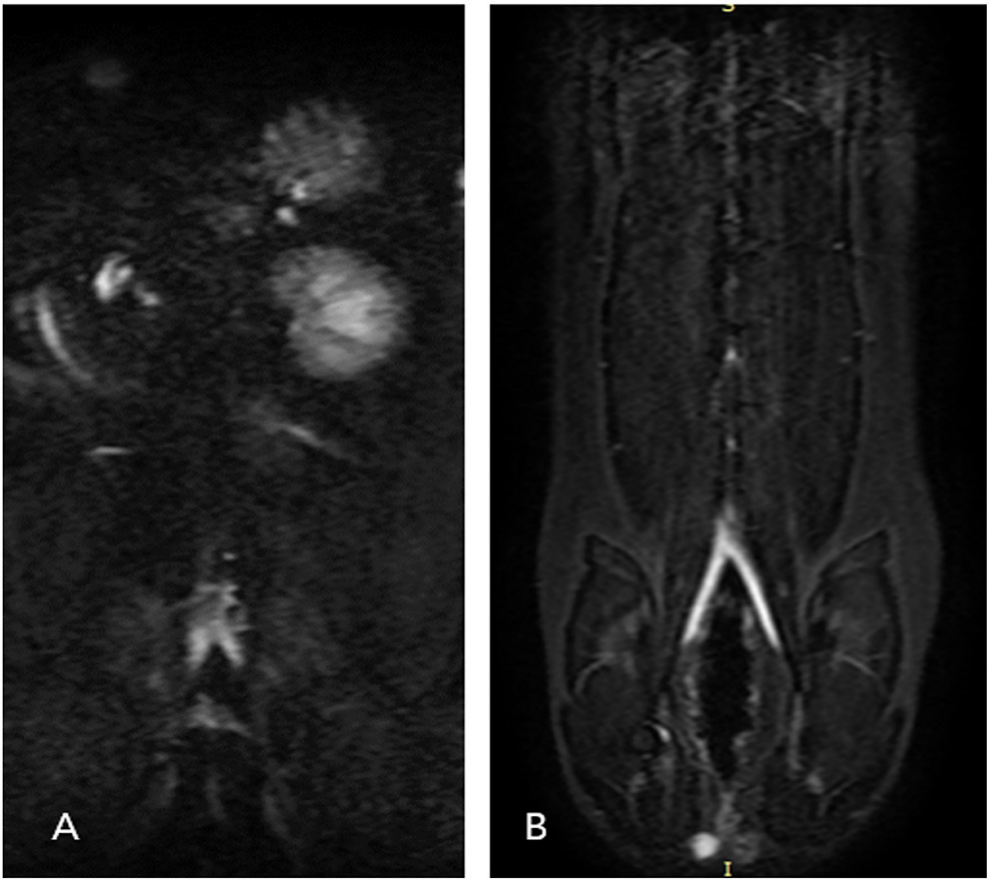
Dorsal single-shot fast spin-echo (SSFSE) images for cat 6 **(A)** and cat 8 **(B)**. Single-shot two-dimensional acquisition, which is a prerequisite for performing 3D ECG-FSE, was not achieved because a clean peripheral pulse gating waveform could not be obtained due to the fast heart rate of cat 6. However, single-shot 2D acquisition was normally achieved for cat 8. The 3D ECG-FSE images of D and F are compared in Figure 5. 3D ECG-FSE, three-dimensional echocardiograph-gated fast spin echo magnetic resonance angiography; SSFSE, single-shot fast spin-echo.

All eleven cats underwent NE-MRA using both 3D ECG-FSE and 3D TOF techniques. The average scan times of 3D TOF and 3D ECG-FSE were 524 (508–528) s and 641.2 (559–762) s, respectively. However, an additional dorsal single-shot fast spin-echo (SSFSE) sequence was required to determine the optimal trigger delays in 3D ECG-FSE MRA (Figure 3). Consequently, the total average scan time using 3D ECG-FSE was 1024.3 s (879–1,255) s (Table 4).

**Table 4:** General anesthesia variables for 3D ECG-FSE and 3D TOF.

**Subject**	**MRA**	**Heart rate (beats per minute)**	**Systolic blood pressure (mmHg)**	**Respiratory rate (breaths per minute)**	**Acquisition time (minutes:seconds)**
Cat 1	3D ECG-FSE	120	100	8	10:25
	3D TOF	110	100	8	08:48
Cat 2	3D ECG-FSE	112	112	8	11:10
	3D TOF	106	106	8	08:42
Cat 3	3D ECG-FSE	93	90	9	12:42
	3D TOF	97	90	9	08:31
Cat 4	3D ECG-FSE	108	91	7	10:31
	3D TOF	100	83	7	08:28
Cat 5	3D ECG-FSE	94	80	10	10:17
	3D TOF	90	80	10	08:48
Cat 6	3D ECG-FSE	N/A	80	12	01:48
	3D TOF	N/A	80	12	08:48
Cat 7	3D ECG-FSE	88	73	9	10:03
	3D TOF	89	80	9	08:48
Cat 8	3D ECG-FSE	80	80	9	09:19
	3D TOF	90	88	9	08:48
Cat 9	3D ECG-FSE	114	97	8	11:18
	3D TOF	95	90	8	08:48
Cat 10	3D ECG-FSE	98	115	10	10:47
	3D TOF	87	80	10	08:48
Cat 11	3D ECG-FSE	89	85	8	10:20
	3D TOF	100	100	8	08:48

*N/A, not available; 3D ECG-FSE, three-dimensional echocardiograph-gated fast spin echo; 3D TOF, three-dimensional time-of-flight; MRA, magnetic resonance angiography*.

A total of 22 lower limbs and 33 anatomical arterial segments were quantitatively evaluated using rSI in 11 cats and qualitatively evaluated using IQS in 10 cats. The 3D ECG-FSE MRA images of one cat were excluded because of poor image quality caused by a non-detectable pulse gating signal. During the quantitative analysis, SNR could not be calculated because the parallel imaging technique, which resulted in a heterogeneous noise distribution, was used as an option for the 3D TOF protocol (23). rSI values were measured at 33 arterial segments of the aorta and external iliac arteries in 11 cats using 3D ECG-FSE and 3D TOF.

### Comparison of Imaging Parameters of 3D ECG-FSE and 3D TOF MRA

**Figure 4:**
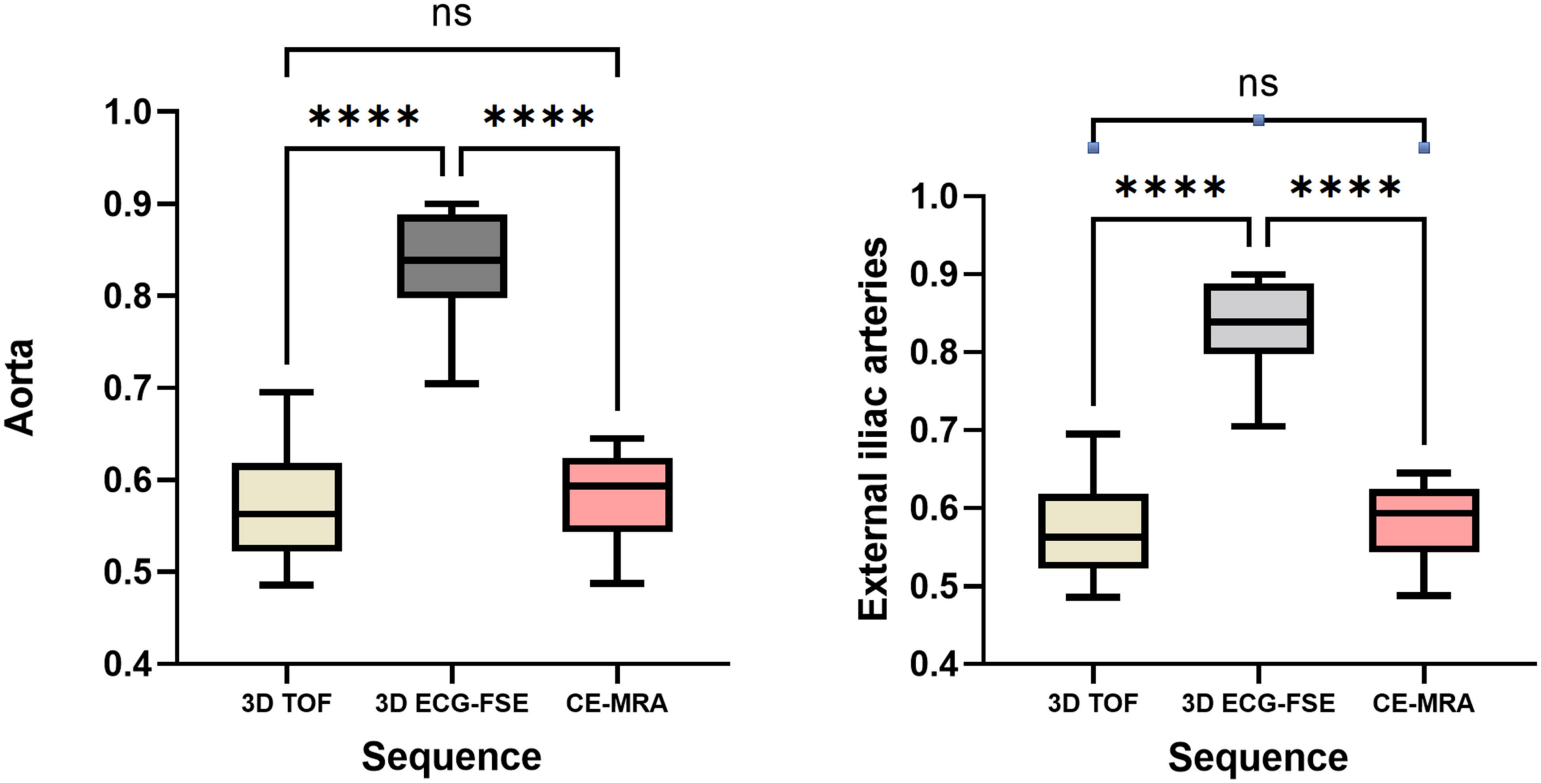
Box and whisker plots of rSI for the aorta and external iliac arteries measured using 3D ECG-FSE, 3D TOF and CE-MRA for clinically healthy cats. Boxes indicate the sample variation, and whiskers represent the standard deviation. It was confirmed that the rSI of 3D ECG-FSE was significantly higher than that of 3D TOF and CE-MRA for all arterial segments. Although the rSI of 3D TOF was lower than that of CE-MRA for aorta and external iliac arteries, however there was no significant difference. ****Statistically significant difference (*P* < 0.001) determined by an analysis of variance followed by the one-way ANOVA. rSI, relative signal intensity; 3D ECG-FSE, three-dimensional echocardiograph-gated fast spin echo; 3D TOF, three-dimensional time-of-flight; FSPGR, contrast-enhanced 3D TOF fast spoiled gradient-echo; ns, no significance.

The rSI values of 3D ECG-FSE MRA, 3D TOF, and CE-MRA for the aorta were 0.83 ± 0.06, 0.57 ± 0.06, and 0.58 ± 0.05 respectively. The rSI values of 3D ECG-FSE MRA, 3D TOF, and CE-MRA for the external iliac arteries were 0.80 ± 0.05, 0.45 ± 0.06, and 0.57 ± 0.03, respectively. The rSI for all segments observed using 3D TOF were significantly lower than those observed using 3D ECG-FSE (*P* < 0.001) and similar to those observed using CE-MRA. The highest segment rSI was observed in the aorta using 3D ECG-FSE (Figure 4).

### Quantitative Image Analysis

**Figure 5:**
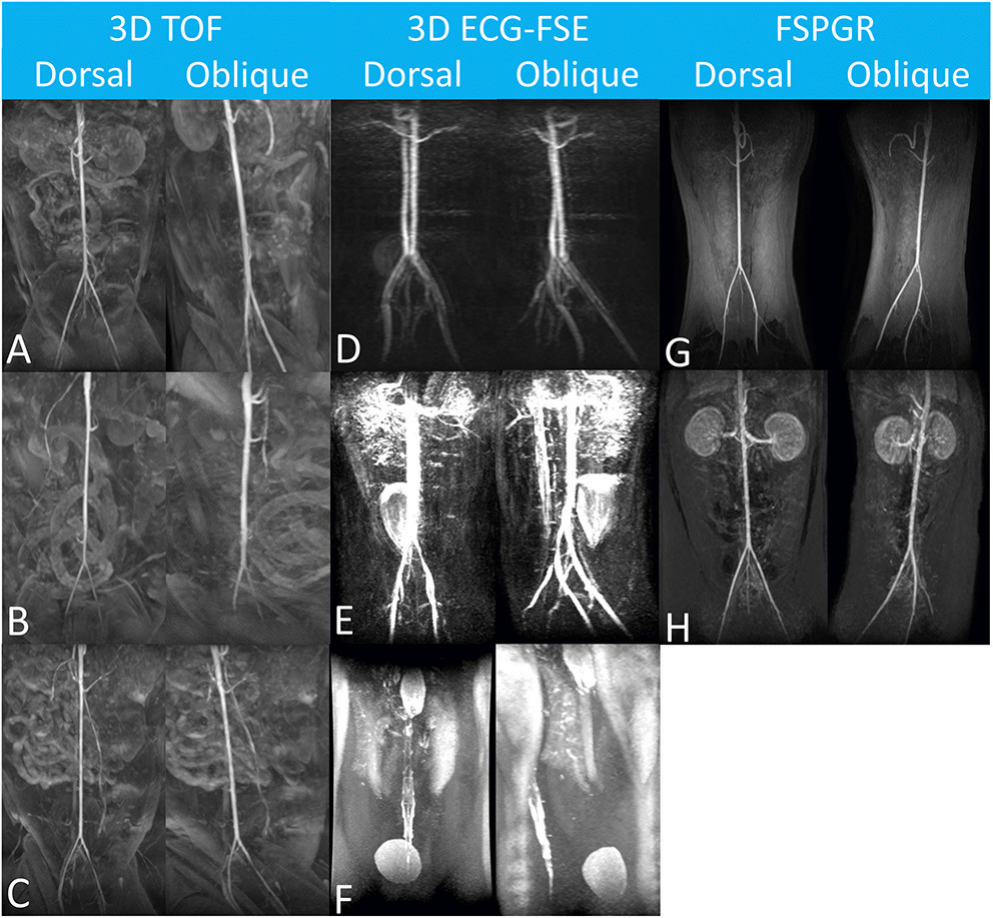
Maximum-intensity projection reconstruction of magnetic resonance angiography images of each dorsal and oblique views obtained using 3D TOF **(A–C)**, 3D ECG-FSE **(D–F)** and FSPGR. The aorta and external iliac arteries were excellently delineated with no venous contamination and were scored as “Excellent” **(A,B,G)**. In **(C,D,H)**, it was scored as “Good” due to minor venous contamination. Considering the margin of the arterial wall, the venous contamination overlapping the aorta and external iliac arteries, and the overall diagnostic qualities, image reconstruction was scored as “Fair” **(E)**, and “poor” **(F)**, respectively. N/A, not available; 3D ECG-FSE, three-dimensional echocardiograph-gated fast spin echo; 3D TOF, three-dimensional time-of-flight; FSPGR, contrast-enhanced 3D TOF fast spoiled gradient-echo.

The subjective IQS for the aorta were rated as either “excellent” (10/11, 91%) or “good” (1/10, 9%) using 3D TOF, and as “good” (3/11, 27%) using 3D ECG-FSE. The subjective IQS for the external iliac arteries were rated as either “excellent” (10/11, 91%) or “good” (1/10, 9%) using 3D TOF, and as “good” (2/11, 18%) using 3D ECG-FSE (Table 5). A non-diagnostic image was obtained when 3D ECG-FSE was used (Table 5 and Figure 5).

**Table 5:** Image quality assessment of the aorta and external iliac arteries.

**Anatomic location**	**Sequence**	**Score**			
		**Excellent**	**Good**	**Fair**	**Poor**
Aorta	3D TOF(n)/3D FSE(n)/FSPGR(n)	10/0/5	1/3/5	0/7/0	0/1/0
External iliac arteries.	3D TOF(n)/3D FSE(n)/FSPGR(n)	10/0/4	1/2/6	0/8/0	0/1/0

*3D ECG-FSE, three-dimensional echocardiograph-gated fast spin echo; 3D TOF, three-dimensional time-of-flight; FSPGR, contrast-enhanced 3D TOF fast spoiled gradient echo*.

The IQS for the aorta obtained using 3D TOF (3.95 ± 0.15) were significantly higher than those obtained using 3D ECG-FSE (2.32 ± 0.60) (*P* < 0.001) and similar to those obtained using CE-MRA (3.61± 0.41; using one-way ANOVA). The IQS for the external iliac arteries obtained using 3D TOF (3.98 ± 0.08) were also significantly higher than those obtained using 3D ECG-FSE (2.23 ± 0.56) (*P* < 0.001) and similar to those obtained using CE-MRA (3.57 ± 0.41; using one-way ANOVA).

**Figure 6:**
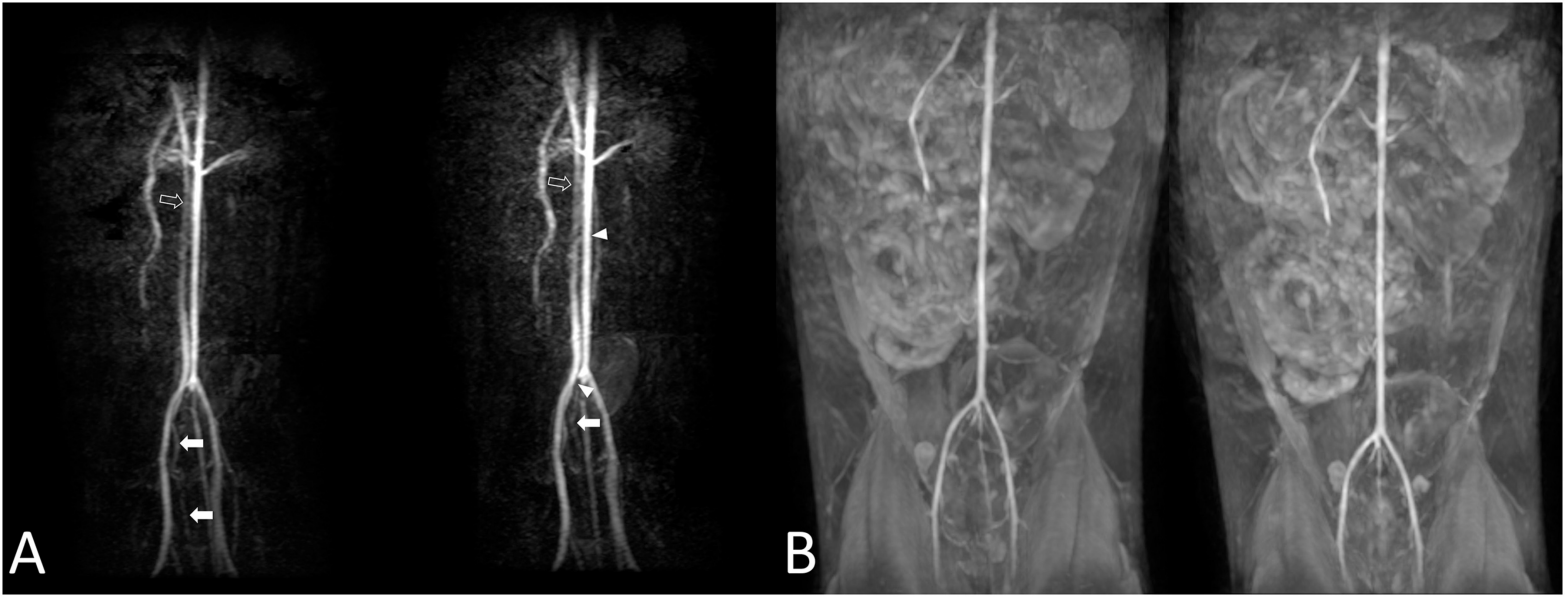
Cat 7. The aorta and external iliac arteries were not the same during the first [**(A)**, left] and second [**(A)**, right] trial using 3D ECG-FSE. Moderate-to-severe venous contamination is observed [**(A)**, arrow], and intermittent filling defect-like artifacts are observed in the aorta [**(A)**, arrowhead]. A visible difference in the diameters **(A)** of the vessels in the aorta and the caudal vena cava was observed [**(A)**, left and right, empty arrow]. The first trial and second trial resulted in observations similar with those obtained using 3D TOF **(B)**. 3D ECG-FSE, three-dimensional echocardiograph-gated fast spin-echo magnetic resonance angiography; 3D TOF, three-dimensional time-of-flight; MRA, magnetic resonance angiography.

When using 3D ECG-FSE in the aorta and external iliac arteries, venous contamination was confirmed in all 11 cats (100%), and filling defect-like artifacts were clearly identified in 8 out of 11 cats (73%) and 9 out of 11 cats (82%), respectively. These artifacts appeared to be signal dropout artifacts, but there was a difference in their degrees. Generally, the arterial vessel margins were blurry and irregular. However, when using 3D TOF, the arterial delineation was excellent and there was little contamination of the veins around the aorta and external iliac arteries (Figures 5, 6).

Repetitive scanning for each sequence to observe the reproducibility and consistency of images indicated no noticeable changes when using 3D TOF. However, changes in blood vessel diameters and artifacts, such as seemingly disconnected blood vessels, filling defects (9 of 11 cats; 81.8%), and loss of blood vessels (3 of 11 cats; 27.3%), were clearly identified when using 3D ECG-FSE. Thus, differences were observed in the reproducibility and consistency of images obtained using the two main NE-MRA techniques (Figure 6).

CE-MRA was performed for 11 cats. Pure arterial contrast images of the aorta (four cats) and external iliac arteries (five cats) were acquired; the remaining cats were confirmed to have venous contamination. CE-MRA was confirmed to have abilities and IQS similar to those of 3D TOF (Figure 5).

### Correlation Between General Anesthesia Variables and Image Quality Scores

No variable correlated with the IQS of 3D TOF in both the aorta and external iliac arteries (heart rate in aorta and external iliac arteries, *P* = 0.516, rs = 0.234; systolic blood pressure in aorta and external iliac arteries, *P* = 0.871, rs = 0.059; respiratory rate in aorta and external iliac arteries, *P* = 0.614, rs = −0.183; and acquisition time in aorta and external iliac arteries, *P* = 0.552, rs = −0.214). Spearman's rank correlation showed moderately negative correlations between the heart rate in the aorta (*P* = 0.017; rs = −0.798), heart rate in the external iliac arteries (*P* = 0.005; rs = −0.830), systolic blood pressure in the aorta (*P* = 0.042; rs = −0.686), systolic blood pressure in the external iliac arteries (*P* = 0.027; rs = −0.709), acquisition time in the aorta (*P* = 0.033; rs = −0.722), acquisition time in the external iliac arteries (*P* = 0.013; rs = −0.779), and the IQS of the 3D ECG-FSE (Table 6). Multiple linear regression analysis (aorta: *F* = 6.471, *p* = 0.033/external iliac arteries: *F* = 5.318, *p* = 0.048) contained four predictors that together accounted for over 70.9% of the variance (adjusted *R*^2^ = 0.709) in image quality score within aorta and for over 65.7% of the variance (adjusted *R*^2^ = 0.657) in image quality score within external iliac arteries. As reported in Table 7, heart rate contributed explaining the image quality score.

**Table 6:** Correlation between variables and image quality scores for 3D ECG-FSE and 3D TOF in general anesthesia.

	**Value**	**Heart rate**	**Systolic blood pressure**	**Respiratory rate**	**Acquisition time**
3D ECG-FSE	r_s_ value	−0.846/−0.675	−0.435/−0.304	0.284/0.041	−0.846/−0.778
(aorta/external iliac arteries)	*P*-value	0.002*/0.032*	0.210/0.393	0.427/0.911	0.002*/0.008*
3D TOF	r_s_ value	0.234/0.234	0.059/0.059	−0.183/−0.183	−0.214/−0.214
(aorta/external iliac arteries)	*P*-value	0.516/0.516	0.871/0.871	0.614/0.614	0.552/0.552

*Spearman's correlation coefficient (rs value) and statistical significance (P-value) for correlations between variables in general anesthesia and image quality scores for 3D ECG-FSE MRA and 3D TOF*.

*Statistically significant. An alpha level of 0.05 was used to determine statistical significance.

*3D ECG-FSE, three-dimensional echocardiograph-gated fast spin echo; and 3D TOF, three-dimensional time-of-flight*.

**Table 7:** Multiple linear regression analysis for aorta and external iliac arteries between variables and image quality scores for 3D ECG-FSE in general anesthesia.

	**Variables**	**95% confidence interval**	**β**	***p***
3D ECG-FSE (aorta/external iliac arteries)	Heart rate	(−0.074, −0.009)/(−0.062, −0.004)	−0.976/−0.923	0.021/0.034
	Systolic blood pressure	(−0.021, 0.036)/(−0.020, 0.032)	0.189/0.180	0.526/0.575
	Respiratory rate	(−0.408, 0.250)/(−0.433, 0.161)	−0.161/−0.333	0.565/0.292
	Acquisition time	(−0.008, 0.005)/(−0.008, 0.004)	−0.144/−0.214	0.645/0.531

*F = 6.471/5.318, P = 0.033/0.048, R^2^ = 0.838/0.900; adjusted R^2^ = 0.709/0.810*.

*β, standardized coefficients; 3D ECG-FSE, three-dimensional echocardiograph-gated fast spin echo*.

### Inter-observer Agreement

Inter-observer agreement for rSI and IQS in the aorta and external iliac arteries was assessed. Excellent agreement was achieved for rSI between the two observers using the two main NE MRA techniques, as determined via the intraclass correlation coefficient test (3D ECG-FSE in aorta, r = 0.999; 3D ECG-FSE in external iliac arteries, r = 0.994; 3D TOF in aorta, r = 0.956; and 3D TOF in external iliac arteries, r = 0.955; CE-MRA of the aorta, r = 0.965; and CE-MRA of the external iliac arteries, r = 0.938). Regarding IQS, an almost perfect agreement was confirmed between the two observers for 3D ECG-FSE in the aorta (r = 0.916), 3D ECG-FSE in the external iliac arteries (r = 0.996), 3D TOF in the aorta (r = 1.000), 3D TOF in the external iliac arteries (r = 0.809), CE-MRA of the aorta (r = 0.890), and CE-MRA of the external iliac arteries (r = 0.991). The measurements obtained by the two readers showed high inter-observer agreement, thereby indicating the reproducibility of these measurements.

### Cross Analysis

A Fisher's exact test was conducted to determine the relationship between sequence and IQS. As an expected frequency of <5 accounted for 5(56%) of 9 cells, the accuracy of Fisher's exact test, not the Pearson chi-square test, was identified. The sequence and IQS were related to each other (*P* < 0.001) (Tables 8, 9).

**Table 8:** Fisher's exact test between MRA and image quality scores in the aorta.

	**Imaging quality score**			**Fisher's exact test (*P*)**
	**Excellent**	**Good**	**Fair**	
3D TOF	10	1	0	<0.001
3D ECG-FSE	0	2	8	
FSPGR	5	5	0	
Total	15	8	8	

*MRA, magnetic resonance angiography; 3D ECG-FSE, three-dimensional echocardiograph-gated fast spin echo; 3D TOF, three-dimensional time-of-flight; FSPGR, contrast-enhanced 3D TOF fast spoiled gradient echo*.

**Table 9:** Fisher's exact test between MRA and image quality scores in the external iliac arteries.

	**Imaging quality score**			**Fisher's exact test (*P*)**
	**Excellent**	**Good**	**Fair**	
3D TOF	10	1	0	<0.001
3D ECG-FSE	0	3	7	
FEPGR	4	6	0	
Total	10	4	7	

*MRA, magnetic resonance angiography; 3D ECG-FSE, three-dimensional echocardiograph-gated fast spin echo; 3D TOF, three-dimensional time-of-flight; FSPGR, contrast-enhanced 3D TOF fast spoiled gradient echo*.

## Discussion

In cats with cardiomyopathies, the diagnosis of FATE using abdominal ultrasonography requires technical expertise, and it may be difficult to scan the thrombi when they are small or located in the peripheral arteries. Advanced imaging modalities such as CTA and CE-MRA can be applied; however, adverse effects may occur with the use of contrast media, as reported previously (24, 25). No previously published veterinary medicine study has reported the use of NE-MRA to visualize the aorta and external iliac arteries in cats. The findings of this study demonstrated that NE-MRA can be used to visualize the aorta and external iliac arteries in clinically healthy cats without the use of contrast media. This can thus provide anatomical information and support FATE diagnosis.

Three important findings were observed in this study. First, TOF is one of the oldest NE-MRA techniques and is rarely used for lower-extremity peripheral vascular diseases because other protocols, such as quiescent-interval signal-shot (QISS), provide better image quality (26). Instead, non-subtractive, inflow-dependent 3D TOF methods are widely used to evaluate the extracranial carotid arteries and circle of Willis due to its isotropic resolution in humans. Moreover, TOF is mainly used for the evaluation of brain arterial diseases in veterinary medicine (27, 28).

However, 3D TOF is prone to artifacts from flow saturation in the horizontal direction due to the rapid and repeated application of the excitation RF pulse and tracking of the venous saturation of the RF pulse, which is typically placed just downstream of the imaging region (26). To compensate for this, vein contamination was prevented by adding a fat saturation band in the inferior direction at the location where the vein descended from the heart. The intravascular mottled artifacts due to a signal dropout were subsequently reduced by providing overlapping in the slab. In this study, 3D TOF had excellent image quality, less venous contamination, and better arterial delineation than 3D ECG-FSE.

Furthermore, a common disadvantage of TOF is the long scanning time in humans. However, in our study, the average scanning time of 3D TOF was ~9 min shorter than that of 3D ECG-FSE. It is because 3D TOF required only post-processing after the sequence, while 3D ECG-FSE needs optimal SSFSE sequence and an additional processing step to determine the optimal trigger delays. Also, scanning time of 3D ECG-FSE can be prolonged depending on cat size. Additionally, 3D TOF had high consistency and reproducibility compared with 3D ECG-FSE when visualizing the aorta and external iliac arteries in healthy cats.

Second, 3D ECG-FSE is known as an excellent NE-MRA technique that allows visualization of the lower arteries without motion artifacts in humans (10, 19, 20). However, in the current study, the image quality obtained using 3D ECG-FSE to visualize the aorta and external iliac arteries in clinically healthy cats was not as good as that obtained using 3D ECG-FSE in humans.

In principle, 3D ECG-FSE involves the voxel-wise subtraction of two 3D datasets of fast arterial flow during the systole and slow venous flow during the diastole obtained through ECG gating or pulse gating. Consequently, the signals of the veins and surrounding tissues are eliminated, and only the signals of the arteries are obtained (17). However, when the difference in the arterial signals of the systole and diastole is minimal, poor arterial contrast images are obtained. Sensitivity to cardiac arrhythmia and spatial blurring due to T2 decay during rapid FSE echo training also results in contaminated venous images (9, 16).

Generally, the heart rate of cats is approximately 100 beats per minute during anesthesia (just before scanning), which is higher than the 60 bpm average in humans (29, 30). Nevertheless, a heart rate of approximately 100 bpm is still too fast to capture an image of a small subject such a cat. According to a previous study in humans, the image quality scores for subjects with heart rates ≤ 65 bpm were significantly better than those for subjects with higher heart rates (31).

In this study, a fast and shallow peripheral pulse was confirmed during pulse monitoring, despite general anesthesia. This made it difficult to achieve sufficient relaxation and contraction during cardiac cycles and resulted in the degraded quality of the images with venous and surrounding tissue contamination and artifacts. The IQS of 3D ECG-FSE had a moderately negative correlation with the heart rate, systolic blood pressure, and scan time (Table 6).

Technical problems could be considered the cause of artifacts and noise in 3D ECG-FSE. The bandwidth of 3D ECG-FSE, which is the range of frequencies involved in the transmission or reception of an electronic signal, had to be higher than that of lower-extremity MRA in humans to acquire more systolic and diastolic data of remarkably fast cardiac cycles in cats. The disadvantage of a higher bandwidth is a larger amount of noise and lower SNR owing to the larger frequency range (32). Consequently, the possibility that the high bandwidth caused more artifacts in the 3D ECG-FSE image was also considered.

Another drawback of 3D ECG-FSE is that it requires additional time to obtain a preparatory single-shot two-dimensional acquisition sequence to find the specific trigger delays for the systole and the diastole (16, 17). Each single-shot 2D acquisition is performed with two or three RR intervals to produce separate images for each phase in humans (16, 33, 34). However, the cats in this study required at least 8–12 RR intervals because of their shallow cardiac rhythm. Additional 2–4 min were required to obtain the SSFSE sequence for one trial. If the sequence is not acquired immediately, more time is required for repeated scans. If specific trigger delays could not be acquired despite repeated retakes, then the image quality of 3D ECG-FSE was poor (Figures 3, 5). The longer the RR interval, the longer the acquisition time, and the lower the image quality (Table 6).

Third, our findings showed that a high signal strength did not indicate good image quality for the aorta and external iliac arteries when using NE MRA. The rSI of 3D ECG-FSE was significantly higher for all segments, including the aorta and external iliac arteries, compared with those of 3D TOF and CE MRA. It was confirmed that rSI does not have a significant effect on image quality.

Based on the complete occlusion of the middle cerebral artery observed through 3D TOF in a previous study that induced canine ischemic stroke model, we inferred that arterial thromboembolism occurring in feline aorta and external iliac arteries could be visualized using 3D TOF (35). However, since this study involved only clinically healthy cats, it is not known whether artifacts that can mimic thrombi, which is one of the common disadvantages of 3D TOF, will be noticeable in actual patients with FATE and whether there could be better results with 3D ECG-FSE than with 3D TOF sequences. Further studies comparing the image quality of 3D TOF and 3D ECG-FSE in cats with FATE are warranted to evaluate their clinical significance.

Regarding the overall condition of cats with FATE, the application of contrast medium and long-term general anesthesia can further worsen the patient's condition. In contrast, when using 3D TOF as a diagnostic tool in cats with FATE, adverse effects caused by contrast media could be avoided.

There were some limitations to this study. First, our sample size was small, and the statistical power was insufficient to detect associations. Although all 11 cats were scanned simultaneously with administration of the contrast agent, the contrast patterns were different for each cat. We confirmed that approximately half of the cats were in the venous phase before the arteries filled. Therefore, the average IQS obtained via CE-MRA was slightly lower than that obtained via 3D TOF, although this difference was not statistically significant.

Moreover, the dose and injection rate of the contrast medium and the contrast agent tracking methods used in this study were applied based on the guidelines stipulated for humans. It was difficult to modify and apply the fluoroscopic trigger method to detect the contrast agent peak in the abdominal aorta of the evaluated cats due to their smaller body size and higher heart rate compared with those of humans. Therefore, it is necessary to study the CE-MRA protocol for evaluation of arteries in the lower extremities of cats specifically.

## Conclusion

The ability of NE-MRA to visualize the feline aorta and the external iliac arteries was examined in this study. Unlike human medicine, 3D ECG-FSE was confirmed to result in the deterioration of image quality (venous contamination, artifacts, and decreased arterial delineation) in cats, and repetitive and reproducible images were not obtained because of their fast and shallow cardiac cycle. 3D TOF is more reliable and reproducible than the 3D ECG-FSE technique because it results in good image quality that is sufficient for visualizing the aorta and external iliac arteries in clinically healthy cats. Therefore, it is expected to be used in the future to diagnose thromboembolism, assess disease severity, choose appropriate therapy, and determine the treatment progress and prognosis of patients with FATE.

## Data Availability Statement

The datasets presented in this study can be found in online repositories. The names of the repository/repositories and accession number(s) can be found in the article/supplementary material.

## Ethics Statement

The animal study was reviewed and approved by CBNUR-1507-21. Written informed consent was obtained from the owners for the participation of their animals in this study. Written informed consent was obtained from the minor(s)' legal guardian/next of kin for the publication of any potentially identifiable images or data included in this article.

## Author Contributions

ML, MK, JisA, JiyA, JY, JC, SO, and DC contributed to the case management. ML wrote the first draft of the manuscript. ML, MK, JisA, JiyA, JC, SO, and DC participated in the revision of the manuscript. All authors have read, commented on, and approved the final manuscript.

## Funding

This work was supported by Institute for Information & communications Technology Promotion (IITP) grant funded by the Korea government (MSIP) (No. 2021-0-00490, Development of Precision Analysis and Imaging Technology for Biological Radio Waves).

## Conflict of Interest

The authors declare that the research was conducted in the absence of any commercial or financial relationships that could be construed as a potential conflict of interest.

## Publisher's Note

All claims expressed in this article are solely those of the authors and do not necessarily represent those of their affiliated organizations, or those of the publisher, the editors and the reviewers. Any product that may be evaluated in this article, or claim that may be made by its manufacturer, is not guaranteed or endorsed by the publisher.
